# Individual and population-level variability in HLA-DR associated immunogenicity risk of biologics used for the treatment of rheumatoid arthritis

**DOI:** 10.3389/fimmu.2024.1377911

**Published:** 2024-05-15

**Authors:** Naonobu Sugiyama, Frances E. Terry, Andres H. Gutierrez, Toshitaka Hirano, Masato Hoshi, Yasushi Mizuno, William Martin, Shin’ichiro Yasunaga, Hiroaki Niiro, Keishi Fujio, Anne S. De Groot

**Affiliations:** ^1^ Rheumatology, Inflammation and Immunology Medical Affairs, Pfizer Japan Inc., Tokyo, Japan; ^2^ EpiVax, Inc., Providence, RI, United States; ^3^ Department of Biochemistry, Faculty of Medicine, Fukuoka University, Fukuoka, Japan; ^4^ Department of Medical Education, Kyushu University Graduate School of Medical Sciences, Fukuoka, Japan; ^5^ Department of Allergy and Rheumatology, Graduate School of Medicine, The University of Tokyo, Tokyo, Japan

**Keywords:** immunogenicity, HLA-DR, T-cell, rheumatoid arthritis, personalized medicine, immunoinformatics

## Abstract

**Hypothesis:**

While conventional in silico immunogenicity risk assessments focus on measuring immunogenicity based on the potential of therapeutic proteins to be processed and presented by a global population-wide set of human leukocyte antigen (HLA) alleles to T cells, future refinements might adjust for HLA allele frequencies in different geographic regions or populations, as well for as individuals in those populations. Adjustment by HLA allele distribution may reveal risk patterns that are specific to population groups or individuals, which current methods that rely on global-population HLA prevalence may obscure.

**Key findings:**

This analysis uses HLA frequency-weighted binding predictions to define immunogenicity risk for global and sub-global populations. A comparison of assessments tuned for North American/European versus Japanese/Asian populations suggests that the potential for anti-therapeutic responses (anti-therapeutic antibodies or ATA) for several commonly prescribed Rheumatoid Arthritis (RA) therapeutic biologics may differ, significantly, between the Caucasian and Japanese populations. This appears to align with reports of differing product-related immunogenicity that is observed in different populations.

**Relevance to clinical practice:**

Further definition of population-level (regional) and individual patient-specific immunogenic risk profiles may enable prescription of the RA therapeutic with the highest probability of success to each patient, depending on their population of origin and/or their individual HLA background. Furthermore, HLA-specific immunogenicity outcomes data are limited, thus there is a need to expand HLA-association studies that examine the relationship between HLA haplotype and ATA in the clinic.

## 1 Introduction

### 1.1 Natural history of RA

Rheumatoid arthritis (RA) is a chronic inflammatory disorder that primarily affects the joints, leading to swelling, pain, stiffness, and gradual joint destruction. It is a global disease with varying prevalence rates in different populations; although estimates suggest that approximately 1% of the world’s population is affected. In the United States and Japan, the prevalence of RA is approximately 0.3-1.0% (1–3). The etiology of RA is multifactorial and results from a complex interplay of genetic, environmental, and hormonal factors.

Among genetic factors, as is true for many autoimmune diseases, specific variants of the human leukocyte antigen (HLA) gene, particularly the HLA-DRB1 alleles, have been strongly associated with RA. This association is more pronounced in certain ethnic populations. Notably, the “shared epitope” (SE) hypothesis postulates that a specific sequence of amino acids in the HLA-DRB1 region is a common feature for most RA patients (4–6). A list of SE alleles can be found in a recent publication by Viatte et al. (7). Other genetic aspects of genetic RA susceptibility are also discussed in the Viatte publication.

More specific examples of differences related to the HLA-DRB1 alleles follow: The HLA-DR*04 allele is frequently found in individuals of European ancestry who have been diagnosed with RA in the United States. Conversely, in the Japanese population, the HLA-DR*09 allele is more commonly associated with RA along with the HLA-DOA gene (see reference (6) for a discussion of these contributors to RA risk). Thus, the prevalence of HLA-DR alleles that are found in native RA patients in the US may differ from the HLA-DR prevalence of native Japanese patients with RA.

These differences in HLA-DR distribution found in RA patient populations may also be relevant to the development of immune responses to RA therapies, since HLA-DR presentation of T cell epitopes derived from therapeutic proteins has been identified as a risk factor for the development of anti-therapeutic antibodies (ATA) (8, 9). RA patients are often treated with biologic protein drugs (also known as biological DMARDs: disease-modifying antirheumatic drugs) that are known to be processed and presented by antigen presenting cells, in the context of HLA-DR molecules, to T cells that can drive ATA responses to the drugs.

Since these ATA can interfere with the efficacy of the biological DMARDs, and HLA-DR-restricted epitopes are the root cause of the ATA, we have hypothesized that regional HLA distributions may help to explain observed differences in immunogenicity (ATA) between global patient groups. In fact, a link between HLA-DR and CD4 T cell activation has already been identified as a factor underlying RA disease activity in studies of patients in Japan (10).

### 1.2 Impact of RA on immune cell populations that can drive ATA

RA also has a direct impact on immune cell populations. T cells, particularly CD4+ T cells, and B cells play key roles in the pathogenesis of RA. They contribute to the chronic inflammation of the joints, and are responsible for the production of autoantibodies, including rheumatoid factor (RF) and anti-citrullinated protein antibodies (ACPAs). In addition, RA patients are noted to have abnormally activated immune cells such as macrophages and dendritic cells, activity (11).

Impaired regulatory T cell responses can also contribute to the development of anti-therapeutic antibodies (ATA) to RA therapies (12, 13). RA patients reportedly have changes to the ratio of T effector helper (Teff) to regulatory T cells (Treg), which can contribute to ATA (14). Effective treatment strategies for RA often target these immune cell populations to reduce inflammation and joint damage (15). The immune system environment is extremely dynamic, and modulatory therapies can have an impact on both local (joint) and systemic (lymphoid system) environments, resulting in changes to joint inflammation and reduction in B cell responses systemically. Likewise, systemic therapies may have an influence on the activation of T helper cells driving ATA. Thus, it is not surprising that effective therapy of RA can also be associated with a reduction in T cell inflammatory responses, an increase in regulatory T cell responses, and a decrease in the inflammatory profile of the immune response which, at the same time, may contribute to a reduction in the anti-therapeutic immune response (ATA) (16).

### 1.3 Biologic DMARDs and JAK inhibitors to treat RA

Biologic therapies for RA known as biologic disease-modifying antirheumatic drugs (bDMARDs) and JAK (Janus kinase) inhibitors have transformed the treatment landscape for rheumatoid arthritis. They work by targeting specific components of the immune system to inhibit the inflammatory processes that are driving inflammation in RA. Readers are referred to an excellent review article by Di Matteo, Bathon, and Emery on therapy for Rheumatoid Arthritis in the Lancet, published in October 2023, for additional information on RA therapy (17). Drugs that are used to treat RA are classified as follows:

#### 1.3.1 TNF inhibitors

These drugs block the cytokine tumor necrosis factor (TNF), which plays a major role in promoting inflammation. Monoclonal antibodies that target TNF include Infliximab (Remicade), Adalimumab (Humira), Certolizumab pegol (Cimzia), and Golimumab (Simponi). Etanercept (Enbrel) is an Fc-fusion of the TNF receptor that also traps TNF, rather than directly inhibiting the cytokine.

#### 1.3.2 Non TNF inhibitors

Both TNFα and IL-6 contribute to inflammation in RA, therefore IL-6 is another inflammatory cytokine that is targeted in RA treatment. IL-6 inhibitors include anti–IL-6 receptor monoclonal antibodies such as Tocilizumab (Actemra) and Sarilumab (Kevzara). Abatacept (Orencia) is a fusion protein comprised of IgG Fc fused to the extracellular domain of CTLA-4, which can bind to the B7 molecules (CD80 and CD86) on antigen-presenting cells. By binding to B7, abatacept prevents a critically important costimulatory signal to T cells, thereby reducing the activity of T cells and the consequent inflammatory response.

#### 1.3.3 JAK inhibitors

RA is also treated using Janus kinase (JAK) inhibitors, a newer class of small molecules (not therapeutic proteins) that block the Janus kinase pathway, which plays a role in the immune response.

### 1.4 Immunogenicity of therapeutic proteins in RA

#### 1.4.1 Clinical observations

Several publications have addressed and reported the incidence and prevalence of ATA in RA. See for example the systemic review by Thomas et al. (18), Woblink et al. (19), and an earlier publication by Garces and Demengeot (20). As discussed above, the recognition and response to these therapeutic proteins is likely heightened in RA due to the underlying dysregulated immune response. For example, approximately 12% of patients treated with therapeutic monoclonal antibodies against TNF develop ATA, but the incidence is much higher in RA patients.

One systemic review found that ATA were involved in decreased response to TNF inhibitors by 27% of patients in RA and by 18% in spondyloarthritis (18). Another systemic review has demonstrated that patients with RA who are treated with TNF inhibitors, such as infliximab or adalimumab, have a higher incidence of developing ATAs compared to those with other inflammatory conditions like Crohn’s disease (13). This propensity to develop ATAs can have important clinical implications, as the presence of these antibodies has been linked to decreased drug efficacy, increased risk of adverse reactions, and reduced treatment durability. Immune response to prescribed RA medication is a problem that affects a significant number of RA patients.

As hypothesized above, the HLA-DR of the individual patient or patient population, as well as to their ability to present natural Treg epitopes may be related to the development of ATA to the individual RA product. This underscores the need for personalized approaches in treating RA, including careful selection of therapeutic agents, taking into consideration the risk of immunogenicity for each individual patient, and monitoring therapeutic response and drug levels over time. Here we focus on populations at the level of geography, but sub populations, disease-specific populations, and individuals may each have different immune responses to therapeutic proteins based on differences in their HLA-DR alleles [Makuch, Van Hamm et al, manuscript in final revision].

#### 1.4.2 Population-level immunogenicity risk assessment with iTEM

To address better understand the influence of HLA distributions on RA therapy, we developed a weighted immunogenicity risk assessment score for populations of patients, that was previously applied to measuring immune responses for individual patients, called the “Individualized T-cell epitope measure” (iTEM) tool. This tool makes it possible to estimate the risk of immune response to a protein antigen based on the HLA-DR frequency in a population, or the combination of HLA-DRs in a single individual (21, 22). The individual score is calculated by counting the number of T effector epitopes, presented by any given HLA-DR that is identified in a monoclonal or DMARD, and adjusted for the presence of validated Treg epitopes (also known as Tregitopes) that are known to occur in monoclonal antibody sequences (8), as described in greater detail below.

Since HLA typing is not routinely performed as an aspect of clinical care for RA patients, we used population-based HLA-DR-adjusted immunogenicity risk assessments to evaluate whether differences in immune responses to biologic products may be related to differences in the HLA prevalence in populations, beginning with HLA prevalence in RA populations in Japan and in the US (to establish an approach that could be used for additional regional populations and sub-populations). iTEM was used to convert HLA-DRB1 allele binding predictions generated by EpiMatrix, an epitope-mapping tool, into an allele-specific scoring system for the HLA distributions observed in Japanese (East Asian) and US (Caucasian) populations. We also identified combinations of HLA-DR alleles for which differences in the predicted immune responses were the greatest (highest risk) or the least (lowest risk).

We then demonstrated that iTEM (HLA-DR-restricted haplotype) analysis of immunogenicity risk appears to differentiate populations in which a specific RA drug may be more likely to activate an immune response and below which immune response is likely to be absent. iTEM may be a useful tool for selecting populations or individuals for which RA drugs may be less likely to elicit ATA, and iTEM may be a useful tool for pre-clinical evaluation of biologic products tailored to selected (different) population groups.

## 2 Methods

### 2.1 Compiling HLA expression frequencies

HLA-DR allele expression frequencies were calculated using gold standard data extracted from The Allele Frequency Net Database (23) with a minimum of four-digit (two field) resolution (e.g., DRB1*01:01). To optimize specificity, population samples were selected based on ethnic origin filters (“Caucasoid” vs. “Oriental” are the terms used in the Database). For the Japanese population, seven population samples with matching ethnic origin (“Oriental”) and geographic filters (“Japan”) were available (**Supplementary Table 1A**). For the Caucasian population, 27 population samples were available across North American and European regions (**Supplementary Table 1B**). Allele frequencies were calculated based on the reported “Total % of individuals that have the allele”, scaled by sample size and aggregated. Alleles expressed at greater than 1% frequency for at least one population were selected (**Table 1**, **Supplementary Table 2**).

**Table 1:** Expression frequency of HLA alleles in Japanese and Caucasian populations.

Allele	HLA Allele Frequency, %	
	Japanese Population	Caucasian Population
DRB1*01:01**	**13.51**	**15.02**
DRB1*01:03	0.00	1.96
DRB1*01:04	**0.00**	**4.02**
DRB1*03:01	0.72	29.04
DRB1*04:01**	**2.28**	**7.82**
DRB1*04:04**	**12.23**	**8.41**
DRB1*04:05**	**25.70**	**6.54**
DRB1*04:08**	**0.92**	**1.94**
DRB1*04:10	**3.80**	**0.09**
DRB1*07:01	0.41	24.05
DRB1*08:01	16.78	3.98
DRB1*08:02	7.38	0.34
DRB1*08:73	3.02	0.01
DRB1*09:01**	28.77	0.71
DRB1*11:01	4.00	11.47
DRB1*11:02	0.20	6.60
DRB1*11:04	4.09	8.29
DRB1*11:58	2.40	0.03
DRB1*12:01	8.93	2.58
DRB1*13:01	1.22	11.39
DRB1*13:02	12.49	7.28
DRB1*13:03**	0.00	2.58
DRB1*14:01	5.10	4.83
DRB1*15:01	13.99	16.04
DRB1*15:02	22.89	1.86
DRB1*16:01**	1.53	6.98

Bold font indicates classical “shared epitope” allele.

**Indicates RA risk allele based on 95% confidence interval of OR>1 (Raychaudhuri et al., 2012).

Details of population frequencies are described in **Supplementary Table 2**.

### 2.2 Compiling observed immunogenicity data for monoclonal antibodies & fusion proteins

A fair estimate of ATA response rate to a given biologic includes clinical data from any available study with significant numbers of systematically chosen participants; however, study size may vary from biologic to biologic and target population to target population. In most cases, an average ATA response rate was calculated based on the rates reported in FDA package inserts using a method described in detail for global population groups in a previous publication by Jawa et al. (24). As previously described, where multiple clinical studies were included, this average was weighted by the number of study participants included for each reported rate. Rates associated with monotherapy were preferred. Where no rates were reported without concomitant medication, a systematic review was performed to justify the inclusion of certain datapoints. Rates associated with very small samples or concomitant medications expected to have significant confounding impacts on ATA response were excluded. Due to measurement inconsistency across product studies, no attempts were made to specify “neutralizing” antibody response rate.

### 2.3 Calculating immunogenic potential scores

Methods to assess the immunogenic potential of a complete protein are available on several public and academic platforms such as the Immune Epitope Database (25), in some cases paired with mathematical models based on hypothetical binding affinities and T cell precursor frequencies (26), or with MAPPs-determined peptidomes (27–29). Here, we used the EpiMatrix scoring system that has been described previously (30, 31). EpiMatrix was developed by De Groot and colleagues at Brown University and licensed to EpiVax in 1998. EpiMatrix and JanusMatrix have been applied and validated in the field of vaccine development, most recently for personalized cancer vaccine development (31)(. Substantial improvements to the EpiMatrix algorithm have resulted in a high degree of accuracy for class II epitopes (77-100%) and higher than 95% for most class I epitopes (32, 33).

Briefly, the EpiMatrix algorithm maps putative ligands to globally representative HLA-DRB1 supertype alleles (34) and calculates a length-normalized score to represent aggregate T cell epitope density. This is called the “Raw” EpiMatrix Score. An adjustment to this score in which the putative ligands specific for known regulatory Tregitopes are excluded from the aggregate calculation has been shown to correlate with the observed immunogenicity of monoclonal antibodies in the clinic (24). This is called the “Tregitope-adjusted” EpiMatrix Score. An adaptation of the EpiMatrix Score for use in personalized medicine is called the individualized T cell Epitope Measure, or “iTEM” Score (21). This score restricts the aggregation of epitope content to a set of two HLA-DR alleles, in order model the scenario of an individual patient, who may be homozygous or heterozygous.

The iTEM Score has been applied to the personalized immunogenicity risk assessment for replacement enzymes (22, 35) and peptides derived from vaccine candidate antigens (36). In previous iterations of iTEM, corrections have been applied for “cross-conservation with self-epitopes” (using the JanusMatrix tool). As this tool has not yet been adjusted for Tregitopes and therefore cannot be applied to antibody-derived biological DMARDs without significant modification, we elected to use the well-standardized Tregitope correction (8) to the EpiMatrix analysis in the models that were applied below (22, 37) instead of the JanusMatrix-corrected version of iTEM (J-iTEM).

## 3 Approach and calculations

### 3.1 Modeling population distributions

To understand the relative immunogenic potential of each biologic specific to distinct populations, we first created 100 iterative random samples of allele frequencies from each population. We used these frequencies to weight the epitope content in each biologic according to the HLA frequency sample, generating an allele frequency-weighted score. The distribution of 100 allele frequency-weighted scores for each biologic for each population was visualized as a violin plot and compared to the conventional EpiMatrix Score based on global HLA supertype alleles (**Supplementary Figure 1**).

### 3.2 Statistical analysis

Medians of Raw and Tregitope-adjusted EpiMatrix Scores by population were compared for each biologic by Wilcoxon signed rank test; p-values <0.05 were considered significant. Results were confirmed with multiple approaches to adjusting p-values for multiple comparisons and quantifying effect sizes (**Supplementary Table 3**).

### 3.3 Modeling risk for individuals in populations

An iTEM Score was calculated for each biologic and each potential combination of HLA alleles in each population. Both “Raw” and “Tregitope-adjusted” iTEM Scores were calculated (**Supplementary Figure 2**).

### 3.4 Differentiation by absolute difference between populations according to joint probability of allele pairs

To compare and visualize the impact of HLA expression frequency on immunogenic risk, box and whisker plots of Tregitope-adjusted iTEM Scores for all potential pairs of HLA alleles were generated. Pairs of alleles with joint probabilities greater than 5%, and absolute differences of greater than 5% between Japanese and Caucasian populations are shown.

## 4 Results

### 4.1 Observed HLA frequencies

Available population data were less abundant for Japanese populations than for Caucasian populations (**Supplementary Tables 1A, B**). Still, sample sizes were sufficient to calculate expression frequencies for multiple common HLA alleles. As shown in **Table 1**, alleles expressed at similar frequencies in both populations include HLA-DRB1*0101 and *1501 (**Supplementary Figure 3**).

Notable differences in the HLA-DR distribution between US and Japanese populations are highlighted here: HLA-DRB1*0901 and *1502 are expressed at high frequency in the Japanese population but not in the Caucasian population, whereas HLA-DRB1*0301 and *0701 are expressed at high frequency in the Caucasian population, but not in the Japanese population. Based on the potential for HLA-DR-restricted T cell epitopes to drive immunogenicity (as measured by ATA), these differences indicate at least some potential for population-specific immunogenic risk based on differential presentation of HLA ligands. A complete, annotated list of evaluated alleles can be seen in **Supplementary Table 2**.

### 4.2 Immunogenicity scores of RA biologics

#### 4.2.1 Range of scores calculated for global supertypes

**Figure 1:**
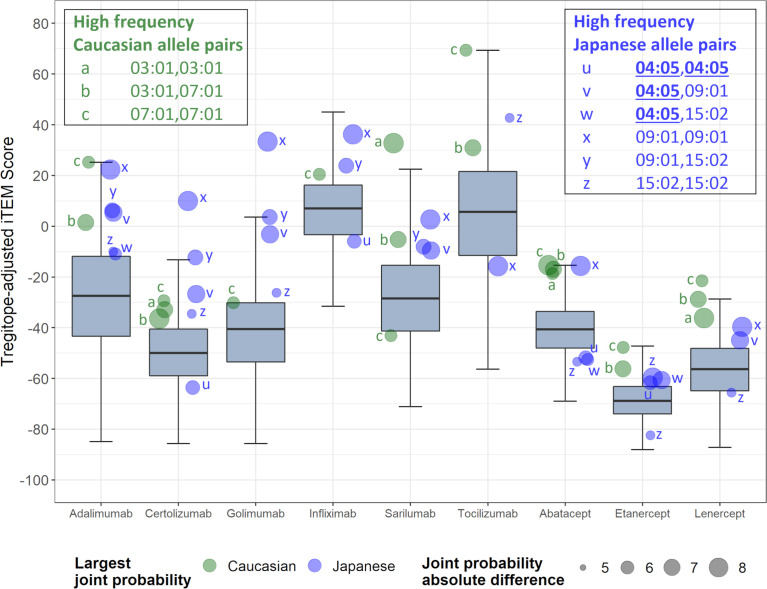
Tregitope-adjusted Immunogenicity Risk Potential Scores of RA Biologics and Benchmark Proteins. The EpiMatrix Tregitope-adjusted Protein Immunogenicity Risk Potential Score represents the aggregate predicted T cell epitope content in each protein, per unit protein length, relative to the expected T cell epitope content in a protein of equivalent length. Proteins with positive scores carry more epitope content than the random expectation, and thereby, increased risk for immunogenic response. Proteins with negative scores carry less epitope content than random expectation, and reduced risk for immunogenic response. These scores are adjusted for the presence of epitopes known to stimulate regulatory T cells, called Tregitopes. Human proteins have a wide distribution of Immunogenicity Risk Potential Scores, whose median is -9.05. The median Immunogenicity Risk Potential Score of secreted human proteins is even lower, at -23.08. Protein Immunogenicity Risk Potential Scores above the median of the human proteome may indicate elevated immunogenic risk for therapeutic protein candidates.

On an overall, global level (not restricted by population-level prevalence data), the Tregitope-adjusted EpiMatrix Immunogenicity Scores of the evaluated RA biologics range from positive 16.99 (Tocilizumab) to negative 60.58 (Etanercept) on the normalized scale illustrated in **Figure 1**. The highest scores are above the average score of a benchmark set of monoclonal antibodies known to simulate ATA in >5% of exposed patients, while the lowest scores are well below the average score of a benchmark set of monoclonal antibodies known to stimulate ATA in <5% of exposed patients (**Figure 1**) (30).

#### 4.2.2 Medians of scores for regional populations

**Figure 2:**
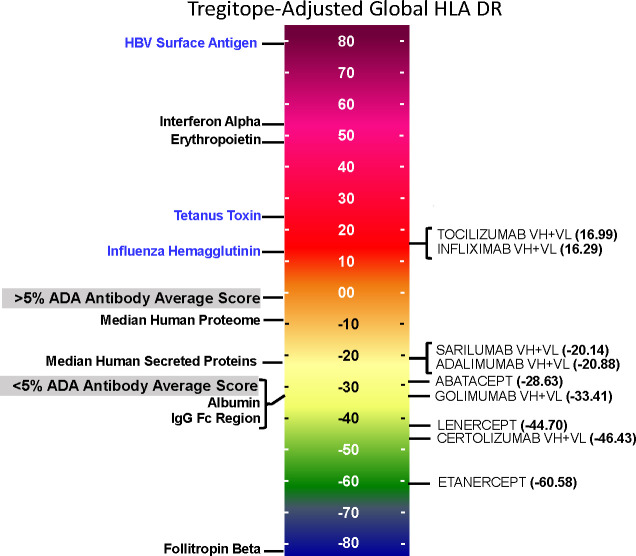
Population-specific EpiMatrix Scores of RA Biologics. We used 100 iterative random samples of allele frequencies from each population to weight the epitope content in each biologic according to the HLA frequency sample, generating an allele frequency-weighted score for the Caucasian (green) and Japanese (blue) populations. Supertype scores (black dot) are not weighted for allele frequency. Raw and Tregitope-adjusted EpiMatrix (EMX) scores were calculated. Applying allele frequency weights to scores reveals variation in distributions by population. In most cases, unweighted scores (calculated using HLA-DRB1 alleles) are higher than frequency-weighted scores.

On a population level, all the medians of the simulated population distributions of Raw EpiMatrix Scores for most RA biologics differ significantly between Japanese and Caucasian populations, except for Sarilumab (**Figure 2**, **Table 2**). Tregitope-adjusted EpiMatrix Score simulated population distribution medians also differ significantly between Japanese and Caucasian populations, with Adalimumab falling near the threshold for significance after adjusting for multiple comparisons (**Supplementary Table 3**). Fusion proteins consistently have the lowest median scores, both Raw and Tregitope-adjusted, but also differ significantly between populations (**Figure 2**). The effect sizes showed that the differences in scores between populations are meaningful except for EpiMatrix scores for Sarilumab and Tregitope-adjusted EpiMatrix for Adalimumab.

**Table 2:** Raw and Tregitope-adjusted EpiMatrix Score distributions for RA biologics.

Biologic	Raw EpiMatrix			Tregitope-adjusted EpiMatrix		
	Median Caucasian	Median Japanese	p-value	Median Caucasian	Median Japanese	p-value
Adalimumab	62.38	61.18	1.48E-08	-20.26	-19.71	0.0027
Certolizumab	20.22	28.05	2.71E-18	-45.28	-40.73	2.71E-18
Golimumab	8.32	10.68	3.76E-18	-38.41	-30.74	2.71E-18
Infliximab	9.34	7.79	2.58E-16	7.84	5.61	4.77E-18
Sarilumab	17.9	18.05	0.265	-19.6	-23.24	2.71E-18
Tocilizumab	41.16	32.01	2.71E-18	12.58	4.27	2.71E-18
Abatacept	-28.83	-30.9	4.23E-18	-37.81	-42.27	2.71E-18
Etanercept	-61.26	-58.66	2.71E-18	-68.01	-67.29	6.80E-15
Lenercept	-44.74	-46.33	2.94E-17	-52.47	-56.25	2.71E-18

#### 4.2.3 EpiMatrix and Tregitope-adjusted scores

As is also shown in **Figure 2**, the unweighted (calculated using supertype HLA-DRB1 alleles) Raw EpiMatrix Scores (not corrected based on Tregitope content) are consistently higher than HLA allele expression frequency-weighted Raw EpiMatrix Scores. After Tregitope-adjustment, unweighted scores for selected DMARDS, specifically Adalimumab, Certolizumab, Golimumab and Sarilumab fall within the distributions of HLA allele expression frequency-weighted scores. In other words, the Tregitope-adjusted score calculated for supertypes is no longer higher than those of the weighted scores for Caucasian and Japanese populations. This result suggests that HLA expression frequencies have differential effects in the immunogenicity risk assessment scores among RA biologics, in particular for those with high Tregitope content. For these biologics, the relationship between potential T effector and Tregitope content is more likely to be affected by variations in HLA expression frequencies.

#### 4.2.4 Impact of population HLA expression frequencies is strongest when Tregitope or T effector epitope content is high

Biologics with high Tregitope content (**Supplementary Table 4**) are more likely to change the Tregitope-adjusted EpiMatrix score/Tregitope content (i.e., T effector/Tregitope) relationship because they have more chances to be affected by HLA frequencies. However, both Tregitope content and potential T effector content can be altered by the HLA frequencies. If the T effector content is lower for one population, and the Tregitope content is identical both populations, differences in the T effector/Tregitope relationship are expected.

#### 4.2.5 Identification of higher risk HLA pairs

**Figure 3:**
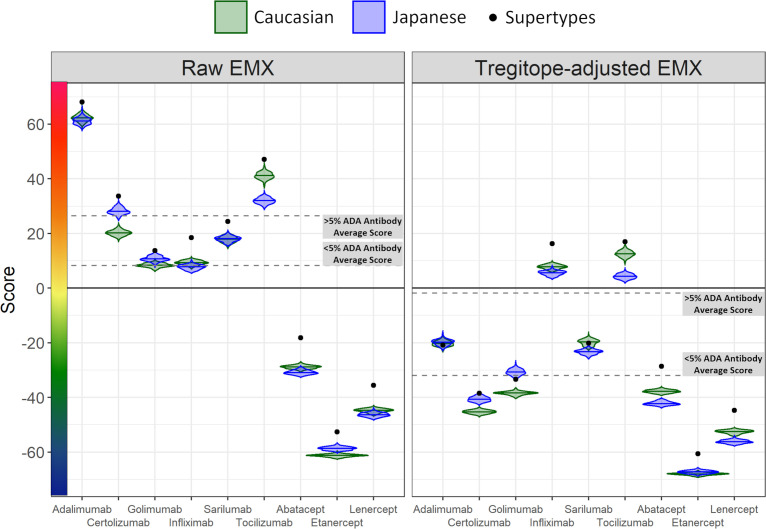
RA biologic patient-specific immunogenic risk varies according to HLA expression frequency in Caucasian and Japanese populations. Figure illustrates the distribution of Tregitope-adjusted iTEM Scores for each biologic. The “box” in the box and whisker plot indicates the second and third quartile of each distribution, separated by a median line, while the “whiskers” indicate the first and fourth quartiles. HLA allele pairs are shown in the colored circles to highlight those pairs which have the greatest difference in joint probability between the Caucasian and Japanese populations. Circles shaded green reflect an allele pair whose joint probability is higher in the Caucasian population than the Japanese population; blue-shaded circles indicate allele pairs whose joint probability is higher in the Japanese population. The size of the circle marker indicates the absolute difference in the joint possibility of the allele pair between the two populations. Only pairs whose absolute joint probability difference is greater than 5% are shown.

Further analysis of pairs of HLA-DR alleles identifies haplotypes that could be ‘higher risk’ in each population, and that may be contributing most to regional differences. Considering the pairs of HLA alleles that might be expressed by individual patients, just three pairs of alleles are expressed at >5% greater joint probabilities in Caucasian populations compared to Japanese populations, while six pairs of alleles are expressed at >5% greater joint probabilities in Japanese populations compared to Caucasian populations (**Figure 3**). Tregitope-adjusted iTEM Scores for the highest differential frequency HLA allele pairs tend to fall in the top quartile of the distributions, especially for the monoclonal antibody biologics, suggesting higher immunogenicity potential for frequently expressed population-specific HLA allele pairs.

## 5 Discussion

To better understand the impact of different HLA distributions in distinct population groups on immunogenicity risk potential of RA therapies, we developed a weighted immunogenicity risk assessment score for populations of patients, and for individual patients, called the “T-cell epitope measure” (iTEM) tool. This tool makes it possible to estimate the risk of immune response to a protein antigen based on HLA prevalence in a population, or in an individual (21).

### 5.1 Summary of key findings

The Human Leukocyte Antigen (HLA) system, specifically the HLA-DR alleles, play a crucial role in the immune response. They are responsible for presenting peptides, including those derived from foreign substances like drugs or pathogens, to the immune system, specifically to CD4+ T cells. The type of HLA-DR allele that is expressed by each individual can influence which peptides are presented to their immune system, which will impact the overall immune response, especially the production of antibodies. HLA-DR differences can also have implications for the generation of anti-therapeutic antibodies (ATAs) to biologic therapeutics. Simply stated, a sequence in each biologic drug might be presented as a foreign peptide by a particular HLA-DR allele that is common in one population, triggering an immune response and ATA production, while the same drug might not trigger the same response in a population where that HLA-DR allele is less common. Geographic variations in HLA-DR alleles have been well documented, reflecting the genetic diversity and evolutionary pressures of different human populations (38).

Here, we have focused on two populations in which similar biological DMARDs are used to treat RA, with potentially different outcomes. We note that HLA-DRB1*09:01 and *15:02 are expressed at high frequency in the Japanese population but not in the Caucasian population, whereas HLA-DRB1*03:01 and *07:01 are expressed at high frequency in the Caucasian population, but not in the Japanese population.

Based on the key contribution of HLA-DR-restricted T cell epitopes to immunogenicity risk potential, these differences indicated at least some potential for population-specific immunogenic risk based on differential presentation of HLA ligands. These differences may be exacerbated in the context of autoimmune diseases such as RA, as certain HLA-DR alleles have been associated the condition. Possessing these specific alleles not only predisposes individuals to RA, but also to a more robust or dysregulated immune response to foreign substances, including biologic therapeutics, which can contribute to increased ATA production.

Differences in the potential immunogenicity risk, based on regional HLA-DR allele differences, are summarized in **Figure 3**. As shown in this figure, on a population level, all the medians of the simulated population distributions of Raw EpiMatrix Scores for most RA biologics differ significantly between Japanese and Caucasian populations, except for Sarilumab.

Take for example, Tocilizumab. Significant differences in the ATA formation to this very important anti-IL-6 therapeutic have been noted in certain populations and could be explained by the fact the HLA-DR*09 allele is highly prevalent among Japanese RA patients. Tocilizumab is known to be associated with limited ATA formation in Japanese patients. The Tregitope adjusted iTEM Scores for DRB1*09:01 homozygous patients fall in the bottom quartile of the distribution for Tocilizumab. In this case, HLA-DRB1*09:01 patients are not expected to develop ATA response to the drug. However, some RA patients in Japan may not carry the HLA-DRB1*09:01 allele that “protects” against ATA for Tocilizumab. In those cases, Fc-fusion proteins such as Abatacept or Etanercept is predicted to be less immunogenic. It is interesting to note that in a previous study, *in vitro* analysis and transcriptomic pathway analysis suggested that a higher frequency of memory CXCR4(+)CD4(+) T cells predicted a better response to CTLA4-Ig (Abatacept) (13). It is not clear whether the memory CD4 T cells in the above study were regulatory T cells, which could explain the observation.

#### 5.1.1 Interpretation of frequency weighted scores, especially iTEM

We evaluated whether the distributions of scores in the violin plots are different between populations. We tried a few tests and found that based on p-values, the populations were different, with EpiMatrix scores for Sarilumab as the only exception. P-values were adjusted for multiple comparisons using 6 different approaches. P-values only tell us whether an effect exists, but do not tell us whether the effect is large enough to be practically meaningful. P-values are influenced by the sample size, so increasing the sample size makes it more likely to find a statistically significant effect, no matter how small the effect truly is in the real world. In contrast, effect sizes are independent of the sample size. For non-parametric tests that used paired samples, effect sizes are calculated using rank-biserial correlations. Categorical effect size interpretations based on criteria defined by different authors were applied, see **Supplementary Table 3**. Only the effect size for Sarilumab EpiMatrix and Tregitope-adjusted EpiMatrix Adalimumab are not classified as large, very strong, or very large. This means that with exception of EpiMatrix scores for Sarilumab and Tregitope-adjusted EpiMatrix for Adalimumab, the scores are significantly different between populations and the differences can be considered meaningful or they suggest practical significance.

#### 5.1.2 Discussion of potential impact of T cell function during treatment

Tregs play a crucial role in maintaining immune tolerance and controlling excessive immune responses. Restoration of regulatory T cell (Treg) function during rheumatoid arthritis (RA) treatment could potentially have a significant impact on disease activity and progression. In the context of RA, their function is often impaired, contributing to the chronic inflammation and tissue damage characteristic of the disease. Enhancing Treg function may not only help manage the symptoms of RA but could also address some of the underlying immune dysregulation driving ATA responses. Some DMARDs have been shown to enhance Treg function (39). The re-activation of regulatory T cell responses may be responsible for some of the “treatment-induced tolerance” that has been observed in many clinical studies (16), and this effect may be more evident for those individuals that carry HLA-DR alleles that are able to present T reg epitopes (Tregitopes), and for DMARDS that contain more Tregitopes.

#### 5.1.3 Consideration of other (non HLA-DR) HLA

Differences in the HLA-DR distributions between Japanese and Caucasian populations are outlined in **Table 1**. Notable differences include HLA-DRB1*01:04, *04:01 and *04:05, all of which are alleles that have a shared amino acid pattern known as the “shared epitope” (**Table 1**). These distinct differences in shared epitope frequency are seen in RA patients from both populations, confirming previous observations that HLA-DR does not directly predict the development of RA. The differences are, however, likely to have an impact on the development of ATA, a hypothesis that is validated in **Table 2** (see significant differences in immunogenicity risk potential, as calculated using EpiMatrix); and in **Figures 2** and **3** as contrasted with **Figure 1**, which compares the relative immunogenicity risk potential of RA therapeutics for global, rather than geographically defined populations.

Other HLA effects such as HLA-DP, DQ, and that of the non-classical DOA HLA gene were not measured in this analysis, for several reasons. Firstly, a significant correlation between ATA and T cell epitope content has been defined previously (24), and this correlation is not preserved when HLA-DP and -DQ predictions are included in the calculation (33). Second, models assessing the impact of the DOA-gene have not been established (6). Additional prospective and retrospective studies may be necessary to define the contributions of alleles beyond HLA-DR.

As can be seen by **Table 1**, the impact of shared epitope alleles on potential for immunogenicity cannot be distinguished from general HLA prevalence frequency in the two populations. Thus, the contribution of SE to differences in immunogenicity risk cannot be quantified in this study.

### 5.2 Advantages and limitations of study

A significant limitation of this study is that it only addresses the risk of immunogenicity in two regional populations – Japanese and American Caucasians. Clearly, there can be significant intra-regional HLA-DR differences in populations (such as can be observed between Caucasian-Americans and African Americans) and there are many global populations for which HLA-DR typing is inconsistent and incomplete. More information on HLA-DR haplotypes is a critical need for improving our understanding of ATA responses to immunomodulatory therapeutics in RA.

Furthermore, while we found that differences in the estimated immunogenicity risk potential that could be associated with the frequency of HLA-DR alleles in each of the regional populations we evaluated to be significant for some of the biological DMARDs, we evaluated relying solely on HLA-DR-associated immunogenicity risk assessment which may be insufficient for predicting anti-therapeutic antibody (ATA) development. This is because ATA formation is a complex process influenced by a multitude of factors, both patient-related and drug-related, and not just by the presence of specific HLA-DR alleles. It is important to note that decreased TCR diversity has been identified in some RA subjects that have the “shared epitope” alleles (40). While we did not find an association between SE and immunogenicity risk in this study, constraints on TCR diversity may have an important impact on ATA responses.

Notably, several GWAS studies have identified a specific HLA-DQ allele (HLA-DQA1*05) as being associated with anti-DMARD antibodies (ATA). In a study of Crohn’s disease subjects, immunogenicity was linked to HLA-DQA1*05 by GWAS for two disparate biologics [adalimumab, and infliximab, (26, 41)]. These two biologic products are significantly different in terms of their protein sequences. A second publication (42), evaluated linkages between ATA to eight different biologics with significantly different mechanisms of actions and protein sequences, and also found a linkage to HLA-DQLA1*05 along with several other HLA-DR alleles (some of which were found to be protective).

Since the correlation with ATA was found irrespective of the sequence of the biologic in these two studies, it is possible that the association with HLA-DQA1*05 is related to a link between the gene and Treg function in the lymphoid follicle, rather than HLA allele restriction of T effector epitopes which are more likely to be found in the CDR regions and less likely to be found in the common framework regions (where Tregitopes are present). An association with Treg function or Tregitopes could also explain linkages to HLA-DRB1*01:01, 03:01, and 07:01 (these are prevalent alleles in European/Caucasian populations) (43). The potential linkages to epitopes (such as Tregitopes) that are conserved between biologics would require further study.

In addition to HLA-DR alleles, other genes involved in the immune response may influence ATA formation, such as genes coding for cytokines and cytokine receptors, T-cell receptors, and B-cell activating factors. Use of other drugs, especially immunosuppressants, can affect the immune response and the risk of ATA development. The presence of aggregates, post-translational modifications, and impurities can also increase the risk of ATA formation. Both the dose and frequency of administration of biological DMARDs can influence the risk of ATA development. Environmental factors, including exposure to pathogens or other foreign antigens, can stimulate the immune system and potentially influence ATA formation. Given the multifactorial nature of immunogenicity, a comprehensive risk assessment for ATA development would need to consider all these factors and their potential interactions, rather than focusing solely on HLA-DR-associated risk.

Lastly, we must address the accuracy of the HLA ligand predictions that are based on EpiMatrix, a tool that has been in continuous use (with updates) since the early 2000’s. In support of the accuracy of this tool, we compiled a retrospective evaluation of EpiMatrix results to internal HLA binding assays which demonstrated that EpiMatrix ranking has a Positive Predictive Value (PPV) of 81% and that the HLA class II predictions were 74% accurate. This study involved more than 1600 assays, performed in house, using the same methodology as published in De Groot et al., 2020 (33).

In addition, for this publication, we performed a high-level analysis of HLA-DR-eluted peptides that have been compiled in the IEDB database (25) to EpiMatrix HLA-DR predictions. We identified 70,594 peptides in the IEDB that were reported (as of March 26, 2024) to have been eluted from human HLA-DR molecules. Using our usual threshold for binding (EpiMatrix Z-score of 1.64), 58,335 (83%) of these peptides contained at least one HLA-allele-specific epitope that is also identified by EpiMatrix. At a slightly lower cutoff that includes “likely” HLA-binding 9-mers (Z-score of 1.28), 64,064 or 91% of the reported eluted peptides contain at least one HLA-allele-specific EpiMatrix ligand (unpublished data analysis by Bill Martin).

Additional T cell epitope and HLA binding validation studies have been published in the course of grant-funded research collaborations, describing T cell immune responses to predicted epitopes *in vitro* using human lymphocytes. For example, 100% of subjects exposed to either Tularemia or Vaccinia responded to pools of T cell epitope clusters that score higher than 20 on the EpiMatrix immunogenicity scale (44–46). In a recent head-to-head comparison, the ClustiMer approach outperformed the standard overlapping peptide approach (usually 15mer peptides overlapping by five amino acids) used by many biologics’ researchers (44). In that comparison, T cell responses to the 15mer overlapping peptides were lower, on average, than the maximal responses induced by the pools predicted using immunoinformatic tools (32).

Overall, the HLA-DR-assessments that are included in this study can be considered to be highly correlated with HLA binding data, HLA ligand elution studies, and T cell assays as currently performed and compiled in public databases.

## 6 Conclusions

In conclusion, analysis of HLA-DR allele haplotypes in rheumatoid arthritis (RA) patient populations could potentially improve the selection of disease-modifying antirheumatic drugs (DMARDs) because these alleles can influence the immune response, including the response to therapeutics. As we have shown here, certain HLA-DR alleles might predispose individuals to a heightened immune response towards specific biologic DMARDs, increasing the risk of developing ATA that can neutralize the drug or accelerate its clearance, thereby reducing their efficacy. Identifying these HLA-DR risk alleles may make possible to select drugs with a lower risk of immunogenicity for these patients. Differences in the frequencies of higher risk HLA pairs in regional populations could also explain any differences in the immunogenicity of biologics that are observed in regional cohorts participating in studies that measure ATA.

In clinical practice, understanding the relationship between HLA-DR alleles and ATA formation could potentially guide personalized therapeutic decisions and the selection of one biological DMARD over another. HLA haplotyping has improved recently, due to the availability of algorithms that deduce HLA haplotype from NGS sequencing of genetic material in peripheral blood (47, 48). Making these decisions will depend on the ability of clinicians to access therapeutic drug monitoring and HLA-DR typing for their patients. In addition, treatment with certain therapeutic agents likely modifies the inflammatory response, leading to the induction of tolerance. Thus, a full understanding of the disease state of the patient, their specific RA-risk factor and phenotype, as well as their HLA-DR allele may be required prior to planning to introduce personalized therapy. More research is needed to fully understand the implications of HLA-DR variations on ATA formation and biologic drug response in different populations.

Achieving the full potential of pharmaceutical products for treatment of Rheumatoid Arthritis (RA) depends on the appropriate selection of the best product for the stage of disease, as well as for the individual patient. Each stage of RA may be phenotypically different, just as each patient may be somewhat genetically unique. Advances have been made in the field of medicine to improve the efficacy of therapy by linking the specific type of therapy by disease characteristic or to stage of disease. Similarly, improvements in RA therapy may be possible if therapy is tailored to characteristics that are unique to populations of patients, and/or to individual patients, based on their individual HLA haplotype and disease phenotype. In other fields, tailored therapy is already being selected. For example, selection of the specific cancer therapy and the design of cancer vaccines can be based on oncogenes that are detected in the patients’ tumors, and on the patient’s HLA alleles (49–51).

## 7 Future directions

This study indicates that HLA-DR genotyping could potentially contribute to the optimization of therapeutic selection. Other factors, such as other genetic factors, the patient’s disease activity and severity, comorbidities, and concomitant medications, should also be considered. Additional prospective studies are needed to support the role of HLA-DR genotyping in guiding biological DMARD selection in clinical practice.

This information could be made available to clinicians who would like to select therapies for their patients that are unlikely to drive ATA. A website devoted to identifying individualized risk of ATA for patients treated with enzyme replacement therapies (Pompe-PIMA) has already been imagined (22). A similar website could also be developed for selecting the best biological DMARD for an individual patient based on their HLA-DR allele haplotype and other genetic factors that are known to be associated with RA. This website could for example take into consideration RA-specific disease states and pre-disposing genetic factors such as mutations associated with regulatory T cell, T follicular helper cell, and cytokine receptor deficiencies (52). One potential use of such a website would be to retrospectively evaluate the association between HLA-DR haplotypes and ATA data generated in the context of clinical trials. A “batch upload” feature was recently added to the PIMA website to facilitate such studies. Both retrospective and prospective studies should be conducted prior to implementing analyses such as PIMA for RA in clinical settings.

## Data availability statement

The original contributions presented in the study are included in the article/**Supplementary Material**. Further inquiries can be directed to the corresponding authors.

## Author contributions

NS: Conceptualization, Funding acquisition, Investigation, Supervision, Writing – review & editing, Data curation, Methodology. FT: Conceptualization, Investigation, Methodology, Formal analysis, Visualization, Writing – original draft. AHG: Writing – review & editing, Data curation, Investigation, Methodology. TH: Writing – review & editing. MH: Writing – review & editing. YM: Writing – review & editing. WM: Writing – review & editing, Investigation, Methodology, Supervision, Validation. SY: Writing – review & editing. HN: Writing – review & editing, Data curation. KF: Writing – review & editing, Conceptualization, Supervision. ADG: Writing – review & editing, Conceptualization, Formal analysis, Funding acquisition, Investigation, Project administration, Supervision, Visualization, Writing – original draft.
