# Supplementary material for: Individual and population-level variability in HLA-DR associated immunogenicity risk of biologics used for the treatment of rheumatoid arthritis
Source: Front Immunol. 2024 May 15;15:1377911. doi: 10.3389/fimmu.2024.1377911 (PMC11134572; doi:10.3389/fimmu.2024.1377911)
Supplement: Supplementary file 1 [file DataSheet_1.docx]

Supplementary Material

**Individual and population-level variability in HLA-DR associated immunogenicity risk of biologics used for the treatment of Rheumatoid Arthritis**

Naonobu Sugiyama*, Frances E. Terry^2*^, Andres H. Gutierrez^2^, Toshitaka Hirano ^1^, Masato Hoshi^1^, Yasushi Mizuno^1^, William Martin^2^, Shin'ichiro Yasunaga^3^, Hiroaki Niiro^4^, Keishi Fujio^5^, Anne S. De Groot^2^

^1^ Pfizer Japan Inc, Tokyo, Japan

^2^ EpiVax, Inc., Providence, RI, USA

^3^ Department of Biochemistry, Faculty of Medicine, Fukuoka University, Fukuoka, Japan

^4^ Department of Medical Education, Kyushu University Graduate School of Medical Sciences, Fukuoka, Japan

^5^ Department of Allergy and Rheumatology, Graduate School of Medicine, The University of Tokyo, Tokyo, Japan

*Currently affiliated with Brown University

* Correspondence: Corresponding Author: [Naonobu.sugiyama@pfizer.com](mailto:Naonobu.sugiyama@pfizer.com)

# Supplementary Figures and Tables

**Supplementary Table 1.** Individual **(A)** Japanese and Caucasian **(B)** populations used to calculate HLA distributions.

**Supplementary Table 1A.**

| **Population** | **Sum of % of individuals that have the alleles in the population^a^** | **Sample Size** |
| --- | --- | --- |
| Japan Hokkaido Wajin | 198.3% | 71 |
| Japan Hyogo | 194% | 32 |
| Japan Nagano | 196.7% | 57 |
| Japan pop 11 | 199.8% | 62 |
| Japan pop 4 | 192.6% | 525 |
| Japan pop 6 | 195% | 129 |
| Japan South | 187.2% | 125 |

^a^Individual HLA-DRB1 alleles were identified in a percentage of individuals in a population. The sum of those percentages per population are shown in the table.

**Supplementary Table 1B.**

| **Population** | **Sum of % of individuals that have the alleles in the population^a^** | **Sample Size** |
| --- | --- | --- |
| Austria | 183.5% | 200 |
| Czech Republic pop 2 | 190.8% | 99 |
| England Bedfordshire | 188.4% | 354 |
| England pop 6 | 189.4% | 177 |
| France Southeast | 183.1% | 130 |
| France West | 147% | 100 |
| Germany Essen | 192.3% | 174 |
| Germany pop 3 | 194.4% | 111 |
| Greece pop 8 | 189% | 83 |
| Greece pop2 | 189.8% | 120 |
| Greece pop3 | 173.5% | 246 |
| Ireland South | 194.8% | 250 |
| Italy Bergamo | 195.9% | 101 |
| Italy North pop 3 | 193.8% | 97 |
| Italy Rome | 195% | 100 |
| Italy Sardinia pop2 | 153.3% | 1129 |
| Netherlands | 191.6% | 447 |
| Netherlands UMCU | 189.4% | 64 |
| Scotland Orkney | 174.2% | 99 |
| Slovenia | 186% | 100 |
| Slovenia pop 2 | 195.5% | 140 |
| Spain (Catalunya, Navarra, Extremadura, Aaragón, Cantabria) | 192.1% | 4335 |
| Spain Barcelona | 191.7% | 941 |
| USA Caucasian Bethesda | 182.7% | 307 |
| USA Caucasian Houston | 184.9% | 194 |
| USA Caucasian Pittsburgh | 198.8% | 177 |
| USA Philadelphia Caucasian | 189.7% | 141 |

^a^Individual HLA-DRB1 alleles were identified in a percentage of individuals in a population. The sum of those percentages per population are shown in the table.

**Supplementary Table 2.** Alleles expressed at greater than 1% frequency in Japanese **(A)** and Caucasian **(B)** populations.

**Supplementary Table 2A. Complete HLA frequency analysis – Japanese population.**

| **Proxy** | **Alleles mapped** | **Allele percentages** | **Sum Percentage** |
| --- | --- | --- | --- |
| DRB1*01:01 | DRB1*01:01 | 12.70 | 13.51 |
|  | DRB1*10:01 | 0.81 |  |
| DRB1*03:01 | | 0.72 | |
| DRB1*04:01 | | 2.28 | |
| DRB1*04:04 | DRB1*04:03 | 4.50 | 12.23 |
|  | DRB1*04:04 | 0.31 |  |
|  | DRB1*04:06 | 7.42 |  |
| DRB1*04:05 | | 25.70 | |
| DRB1*04:08 | DRB1*04:07 | 0.82 | 0.92 |
|  | DRB1*04:08 | 0.10 |  |
| DRB1*04:09 | | 0.10 | |
| DRB1*04:10 | | 3.80 | |
| DRB1*07:01 | | 0.41 | |
| DRB1*08:01 | DRB1*08:03 | 16.78 | |
| DRB1*08:02 | | 7.38 | |
| DRB1*08:04 | | 0.30 | |
| DRB1*09:01 | | 28.77 | |
| DRB1*11:01 | | 4.00 | |
| DRB1*11:02 | | 0.20 | |
| DRB1*12:01 | DRB1*12:01 | 6.92 | 8.93 |
|  | DRB1*12:02 | 2.01 |  |
| DRB1*13:01 | | 1.22 | |
| DRB1*13:02 | | 12.49 | |
| DRB1*13:169 | DRB1*14:01 | 5.10 | |
| DRB1*11:09 | DRB1*14:02 | 0.31 | |
| DRB1*08:73 | DRB1*14:03 | 3.02 | |
| DRB1*11:04 | DRB1*14:05 | 4.09 | |
| DRB1*11:58 | DRB1*14:06 | 2.40 | |
| DRB1*13:21 | DRB1*14:07 | 0.31 | |
| DRB1*15:01 | | 13.99 | |
| DRB1*15:02 | | 22.89 | |
| DRB1*16:01 | DRB1*16:01 | 0.21 | 1.53 |
|  | DRB1*16:02 | 1.32 |  |
| **27 Total** | **33 Total** | **193.36%** | |

**Grey highlight** indicates RA risk allele based on 95% confidence interval of OR>1 *(Raychaudhuri, et al., 2012).* Blue font indicates proxy allele used for in silico prediction based on significant similarity in peptide binding groove residues.

**Supplementary Table 2B.** **Complete HLA frequency analysis – Caucasian population.**

| **Proxy** | **Alleles mapped** | **Allele percentages** | **Sum Percentage** |
| --- | --- | --- | --- |
| DRB1*01:01 | DRB1*01:01 | 13.11 | 15.02 |
|  | DRB1*10:01 | 1.91 |  |
| DRB1*01:03 | | 1.96 | |
| DRB1*01:04 | DRB1*01:02 | 4.00 | 4.02 |
|  | DRB1*01:04 | 0.02 |  |
| DRB1*03:01 | DRB1*03:01 | 29.02 | 29.04 |
|  | DRB1*03:04 | 0.01 |  |
|  | DRB1*03:07 | 0.01 |  |
| DRB1*03:05 | DRB1*03:02 | 0.10 | 0.14 |
|  | DRB1*03:05 | 0.04 |  |
| DRB1*04:01 | | 7.82 | |
| DRB1*04:04 | DRB1*04:03 | 3.01 | 8.41 |
|  | DRB1*04:04 | 5.03 |  |
|  | DRB1*04:06 | 0.37 |  |
| DRB1*04:05 | DRB1*04:05 | 6.53 | 6.54 |
|  | DRB1*04:17 | 0.01 |  |
| DRB1*04:08 | DRB1*04:07 | 1.19 | 1.94 |
|  | DRB1*04:08 | 0.75 |  |
| DRB1*04:10 | DRB1*04:10 | 0.06 | 0.09 |
|  | DRB1*04:11 | 0.02 |  |
|  | DRB1*14:10 | 0.01 |  |
| DRB1*04:13 | DRB1*04:13 | 0.01 | 0.05 |
|  | DRB1*04:37 | 0.04 |  |
| DRB1*07:01 | DRB1*07:01 | 24.03 | 24.05 |
|  | DRB1*07:03 | 0.01 |  |
|  | DRB1*07:08 | 0.01 |  |
| DRB1*08:01 | DRB1*08:01 | 3.61 | 3.98 |
|  | DRB1*08:03 | 0.37 |  |
| DRB1*08:02 | | 0.34 | |
| DRB1*08:04 | | 0.48 | |
| DRB1*08:06 | DRB1*08:06 | 0.10 | 0.55 |
|  | DRB1*08:10 | 0.05 |  |
|  | DRB1*08:12 | 0.01 |  |
|  | DRB1*14:04 | 0.40 |  |
| DRB1*09:01 | | 0.71 | |
| DRB1*11:01 | DRB1*11:01 | 11.43 | 11.47 |
|  | DRB1*11:12 | 0.04 |  |
| DRB1*11:02 | DRB1*04:02 | 3.11 | 6.60 |
|  | DRB1*11:02 | 1.99 |  |
|  | DRB1*11:03 | 1.49 |  |
| DRB1*11:04 | DRB1*11:04 | 8.19 | 8.29 |
|  | DRB1*13:11 | 0.04 |  |
|  | DRB1*14:05 | 0.01 |  |
|  | DRB1*14:23 | 0.05 |  |
| DRB1*11:09 | DRB1*11:15 | 0.04 | 0.80 |
|  | DRB1*11:24 | 0.01 |  |
|  | DRB1*13:05 | 0.59 |  |
|  | DRB1*13:26 | 0.05 |  |
|  | DRB1*14:02 | 0.11 |  |
| DRB1*11:14 | DRB1*04:14 | 0.01 | 0.05 |
|  | DRB1*11:14 | 0.04 |  |
| DRB1*11:58 | DRB1*14:06 | 0.01 | 0.03 |
|  | DRB1*14:17 | 0.01 |  |
|  | DRB1*14:33 | 0.01 |  |
| DRB1*12:01 | DRB1*12:01 | 2.42 | 2.58 |
|  | DRB1*12:02 | 0.16 |  |
|  | DRB1*12:04 | 0.01 |  |
| DRB1*13:01 | DRB1*11:12 | 0.04 | 11.39 |
|  | DRB1*13:01 | 11.30 |  |
|  | DRB1*13:10 | 0.04 |  |
|  | DRB1*13:15 | 0.01 |  |
| DRB1*13:02 | | 7.28 | |
| DRB1*13:03 | | 2.58 | |
| DRB1*13:04 | DRB1*13:04 | 0.07 | 0.11 |
|  | DRB1*14:16 | 0.04 |  |
| DRB1*13:169 | DRB1*14:01 | 2.93 | 4.83 |
|  | DRB1*14:54 | 1.90 |  |
| DRB1*13:21 | DRB1*08:05 | 0.04 | 0.072 |
|  | DRB1*14:07 | 0.04 |  |
| DRB1*08:73 | DRB1*14:03 | 0.01 | |
| DRB1*15:01 | DRB1*15:01 | 15.91 | 16.04 |
|  | DRB1*15:03 | 0.09 |  |
|  | DRB1*15:04 | 0.04 |  |
| DRB1*15:02 | | 1.86 | |
| DRB1*16:01 | DRB1*16:01 | 6.31 | 6.98 |
|  | DRB1*16:02 | 0.65 |  |
|  | DRB1*16:05 | 0.02 |  |
| **35 Total** | **76 Total** | **186.14%** | |

**Grey highlight** indicates RA risk allele based on 95% confidence interval of OR>1 *(Raychaudhuri et al, 2012).* Blue font indicates proxy allele used for in silico prediction based on significant similarity in peptide binding groove residues.

**Supplementary Table 3. Statistical analysis of population EpiMatrix Score distributions.**

| **Biologic** | **EpiMatrix Score** | **P-value adjustment for multiple comparisons** | | | | | | **Effect size** | | | | | |
| --- | --- | --- | --- | --- | --- | --- | --- | --- | --- | --- | --- | --- | --- |
|  |  | **Holm** | **Hochberg** | **Hommel** | **Bonferroni** | **Benjamini & Hochberg** | **Benjamini & Yekutieli** | [**Rank biserial cor (rrb)**](https://easystats.github.io/effectsize/reference/rank_biserial.html) | **Cohen**  **1988** | **Evans  1996** | [**Gignac 2016**](https://www.sciencedirect.com/science/article/abs/pii/S0191886916308194) | [**Funder 2019**](https://journals.sagepub.com/doi/10.1177/2515245919847202) | [**Lovakov 2021**](https://onlinelibrary.wiley.com/doi/abs/10.1002/ejsp.2752) |
| Abatacept | Raw | 4.88e-17 | 3.34e-17 | 2.96e-17 | 7.61e-17 | 6.92e-18 | 2.42e-17 | 0.99 | large | very strong | large | very large | large |
| Abatacept | Treg-adj | 4.88e-17 | 2.71e-17 | 1.91e-17 | 4.88e-17 | 5.42e-18 | 1.89e-17 | 1.00 | large | very strong | large | very large | large |
| Adalimumab | Raw | 4.44e-08 | 4.44e-08 | 4.44e-08 | 2.66e-07 | 1.67e-08 | 5.82e-08 | 0.65 | large | strong | large | very large | large |
| Adalimumab | Treg-adj | 0.005 | 0.005 | 0.005 | 0.05 | 0.003 | 0.01 | -0.34 | moderate | weak | large | large | medium |
| Certolizumab | Raw | 4.88e-17 | 2.71e-17 | 1.91e-17 | 4.88e-17 | 5.42e-18 | 1.89e-17 | -1.00 | large | very strong | large | very large | large |
| Certolizumab | Treg-adj | 4.88e-17 | 2.71e-17 | 1.91e-17 | 4.88e-17 | 5.42e-18 | 1.89e-17 | -1.00 | large | very strong | large | very large | large |
| Etanercept | Raw | 4.88e-17 | 2.71e-17 | 1.91e-17 | 4.88e-17 | 5.42e-18 | 1.89e-17 | -1.00 | large | very strong | large | very large | large |
| Etanercept | Treg-adj | 2.72e-14 | 2.72e-14 | 2.72e-14 | 1.22e-13 | 8.16e-15 | 2.85e-14 | -0.89 | large | very strong | large | very large | large |
| Golimumab | Raw | 4.88e-17 | 3.34e-17 | 2.63e-17 | 6.77e-17 | 6.77e-18 | 2.37e-17 | -1.00 | large | very strong | large | very large | large |
| Golimumab | Treg-adj | 4.88e-17 | 2.71e-17 | 1.91e-17 | 4.88e-17 | 5.42e-18 | 1.89e-17 | -1.00 | large | very strong | large | very large | large |
| Infliximab | Raw | 1.29e-15 | 1.29e-15 | 1.29e-15 | 4.64e-15 | 3.32e-16 | 1.16e-15 | 0.94 | large | very strong | large | very large | large |
| Infliximab | Treg-adj | 4.88e-17 | 3.34e-17 | 3.34e-17 | 8.59e-17 | 7.16e-18 | 2.50e-17 | 0.99 | large | very strong | large | very large | large |
| Lenercept | Raw | 1.76e-16 | 1.76e-16 | 1.76e-16 | 5.29e-16 | 4.07e-17 | 1.42e-16 | 0.97 | large | very strong | large | very large | large |
| Lenercept | Treg-adj | 4.88e-17 | 2.71e-17 | 1.91e-17 | 4.88e-17 | 5.42e-18 | 1.89e-17 | 1.00 | large | very strong | large | very large | large |
| Sarilumab | Raw | 0.27 | 0.27 | 0.27 | 1.0 | 0.27 | 0.93 | -0.13 | small | very weak | small | small | small |
| Sarilumab | Treg-adj | 4.88e-17 | 2.71e-17 | 1.91e-17 | 4.88e-17 | 5.42e-18 | 1.89e-17 | 1.00 | large | very strong | large | very large | large |
| Tocilizumab | Raw | 4.88e-17 | 2.71e-17 | 1.91e-17 | 4.88e-17 | 5.42e-18 | 1.89e-17 | 1.00 | large | very strong | large | very large | large |
| Tocilizumab | Treg-adj | 4.88e-17 | 2.71e-17 | 1.91e-17 | 4.88e-17 | 5.42e-18 | 1.89e-17 | 1.00 | large | very strong | large | very large | large |

^a^P-value adjustment for multiple comparisons and effect size were calculated for Raw and Tregitope-adjusted EpiMatrix scores.

^b^To evaluate whether the effect determined by the p-values was practically meaningful, effect sizes were calculated using rank-biserial correlations. This correlation is applied for non-parametric tests that use paired samples. Values range from -1 complete dominance of the Japanese sample (all values of the Japanese sample are larger than all the values of the Caucasian sample) to +1 complete dominance of the Caucasian sample (all values of the Japanese sample are smaller than all the values of the Caucasian sample). Categorical effect size interpretations based on criteria defined by different authors were determined.

^c^Cohen J. Statistical Power Analysis for the Behavioral Sciences. New York, NY: Routledge Academic. 1988.

^d^Evans, JD. Straightforward statistics for the behavioral sciences. Thomson Brooks/Cole Publishing Co. 1996.

^e^Gignac, GE, Szodorai, ET. Effect size guidelines for individual differences researchers. Personality and Individual Differences. 2016;102.

^f^Funder DC, Ozer DJ. Evaluating Effect Size in Psychological Research: Sense and Nonsense. Advances in Methods and Practices in Psychological Science. 2019;2(2).

^g^Lovakov A, Agadullina ER. Empirically derived guidelines for effect size interpretation in social psychology. Eur J Soc Psychol. 2021;51.

**Supplementary Table 4. HLA frequency-weighted and unweighted (Supertypes) scores**

| **Biologic** | **Raw EpiMatrix score** | | | **Tregitope-adjusted EpiMatrix score** | | | **Tregitope content** | | |
| --- | --- | --- | --- | --- | --- | --- | --- | --- | --- |
|  | **Caucasian** | **Japanese** | **Supertypes** | **Caucasian** | **Japanese** | **Supertypes** | **Caucasian** | **Japanese** | **Supertypes** |
| Adalimumab | 62.85 | 61.74 | 68.17 | -19.86 | -19.39 | -20.88 | 82.71 | 81.12 | 89.06 |
| Certolizumab | 20.67 | 29.03 | 33.67 | -44.79 | -40.16 | -38.48 | 65.46 | 69.18 | 72.15 |
| Golimumab | 8.82 | 11.43 | 13.68 | -38.23 | -30.02 | -33.41 | 47.05 | 41.45 | 47.09 |
| Infliximab | 9.34 | 7.56 | 18.49 | 7.87 | 5.42 | 16.29 | 1.47 | 2.14 | 2.19 |
| Sarilumab | 18.31 | 18.54 | 24.39 | -19.32 | -22.93 | -20.14 | 37.63 | 41.46 | 44.53 |
| Tocilizumab | 41.58 | 32.03 | 47.13 | 13.01 | 4.36 | 16.99 | 28.57 | 27.67 | 30.14 |
| Abatacept | -28.64 | -30.86 | -18.21 | -37.48 | -42.30 | -28.63 | 8.84 | 11.44 | 10.42 |
| Etanercept | -61.28 | -58.61 | -52.66 | -68.01 | -67.31 | -60.58 | 6.72 | 8.70 | 7.93 |
| Lenercept | -44.59 | -46.33 | -35.63 | -52.28 | -56.28 | -44.70 | 7.69 | 9.96 | 9.07 |

The table is colored from high scores in red to low scores in blue.

**Appendix Table** **5.** **References for observed immunogenicity data for monoclonal antibodies & fusion proteins**

| Biologic | Brand | Target | Indication* | Observed ADA, % | Reference |
| --- | --- | --- | --- | --- | --- |
| ADALIMUMAB | HUMIRA | TNFα | Crohn's disease, Ulcerative colitis, Plaque psoriasis, Hidradenitis suppurativa, Non-infectious uveitis | 21.25 | <https://www.accessdata.fda.gov/drugsatfda_docs/label/2021/125057s417lbl.pdf> |
| GOLIMUMAB | SIMPONI | TNFα | Rheumatoid arthritis, Psoriatic arthritis, Ankylosing spondylitis | 31.74 | <https://www.accessdata.fda.gov/drugsatfda_docs/label/2023/125289s150lbl.pdf> |
| INFLIXIMAB | REMICADE | TNFα | Psoriatic arthritis | 27.00 | <https://www.accessdata.fda.gov/drugsatfda_docs/label/2021/103772s5401lbl.pdf> |
| SARILUMAB | KEVZARA | IL-6R | Rheumatoid arthritis | 9.20 | <https://www.accessdata.fda.gov/drugsatfda_docs/label/2023/761037s013lbl.pdf> |
| TOCILIZUMAB | ACTEMBRA | IL-6R | Rheumatoid arthritis | 1.60 | <https://www.accessdata.fda.gov/drugsatfda_docs/label/2022/125276s138lbl.pdf> |
| CERTOLIZUMAB PEGOL | CIMZIA | TNFα | Crohn's disease, Rheumatoid arthritis, Psoriasis | 13.12 | <https://www.accessdata.fda.gov/drugsatfda_docs/label/2022/125160s305lbl.pdf> |
| ABATACEPT | ORENCIA | CD80/86 | Rheumatoid arthritis, Polyarticular juvenile idiopathic arthritis | 2.67 | <https://www.accessdata.fda.gov/drugsatfda_docs/label/2024/125118s249lbl.pdf> |
| ETANERCEPT | ENBREL | TNF | Rheumatoid arthritis, Plaque Psoriasis, Psoriatic arthritis, Ankylosing spondylitis | 6.00 | <https://www.accessdata.fda.gov/drugsatfda_docs/label/2023/103795s5595lbl.pdf> |
| LENERCEPT | NA | TNFα | Rheumatoid arthritis, Multiple sclerosis | 40.00 | https://pubmed.ncbi.nlm.nih.gov/9836378/ |

## *Indications considered to calculate ADA

**Supplementary Figure 1. Modeling population distributions.**

**
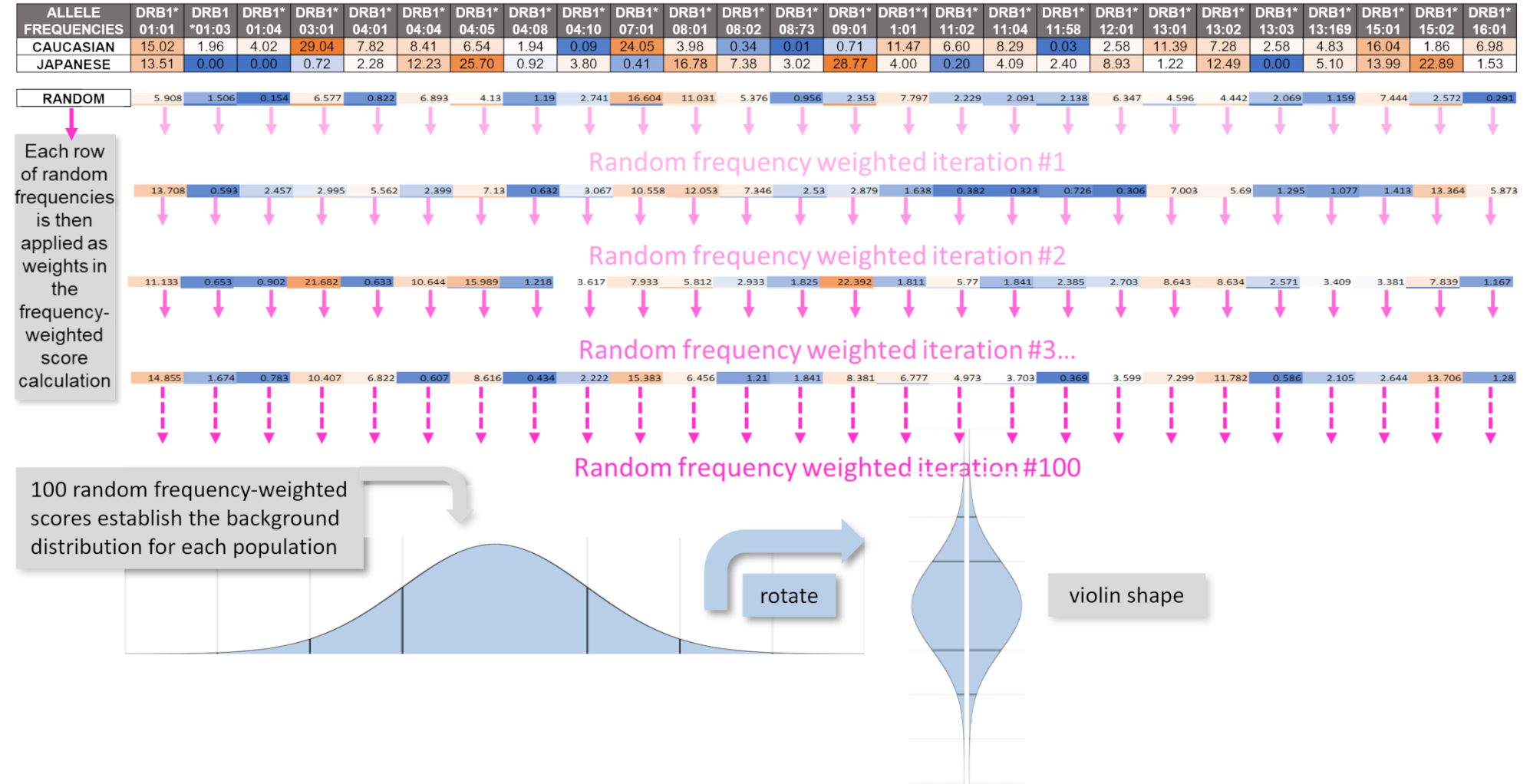
**

To understand the relative immunogenic potential of each biologic specific to distinct populations, we generated 100 random samples of allele frequencies from each population. These frequencies were used to weight the epitope content in each biologic according to the HLA frequency sample, generating an allele frequency-weighted score. The distribution of 100 allele frequency-weighted scores for each biologic for each population was visualized as a violin plot.

**Supplementary Figure 2. Calculation of iTEM Scores & Tregitope-adjusted iTEM Scores.**


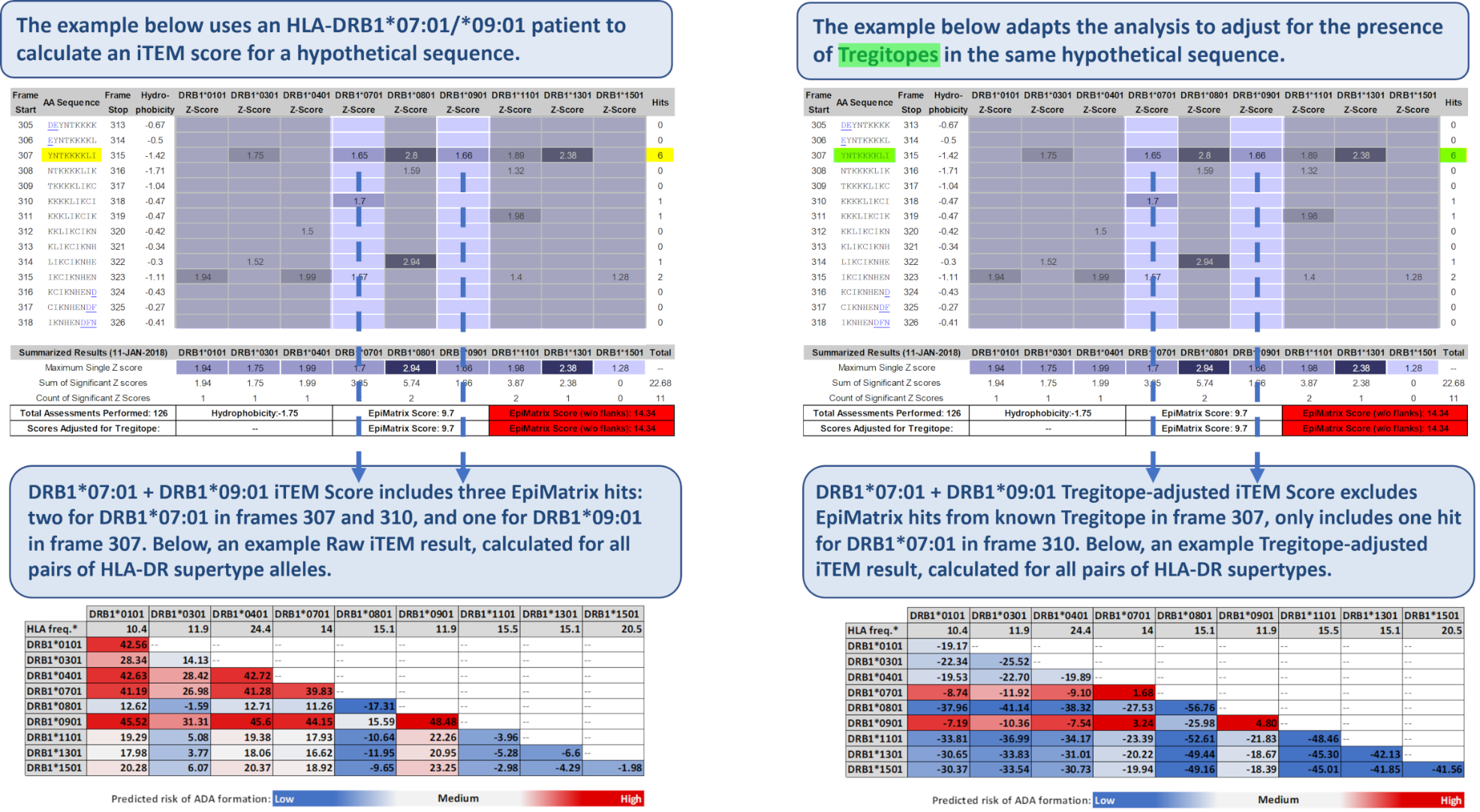


This figure illustrates the calculation of iTEM and Tregitope-adjusted iTEM Scores for one combination of HLA alleles. Input amino acid sequences were parsed into overlapping 9-mers frames. Each 9-mer was then assessed for its binding potential to pairs of HLA-DR alleles. The top 5% of assessments (Z-score>1.64) are considered statistically significant. Z-scores of putative T cell epitopes were considered to calculate iTEM scores. Z-scores of Tregitope 9-mers were excluded from the calculation of Tregitope-adjusted iTEM scores.

**Supplementary Figure 3. Expression frequency of HLA-DR alleles in Japanese and Caucasian populations.**


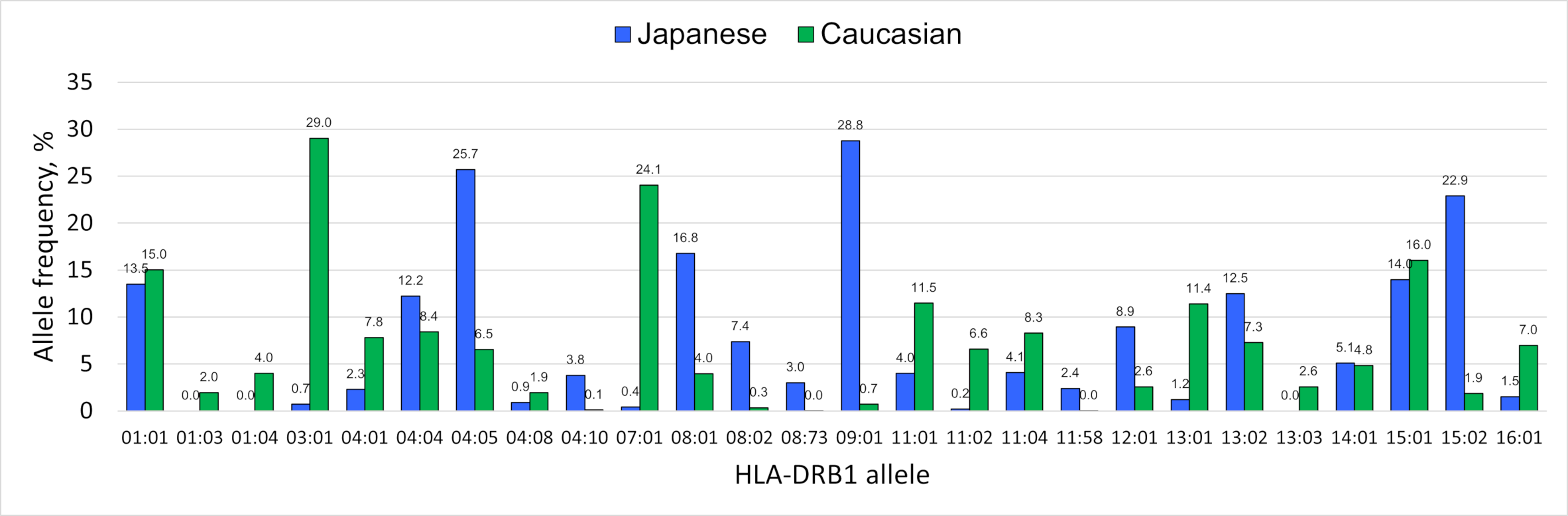


Data from Table 1 is shown as a bar chart.

**Supplementary Figure 4. Immunogenicity Risk Potential Scale with Raw EpiMatrix Scores.**
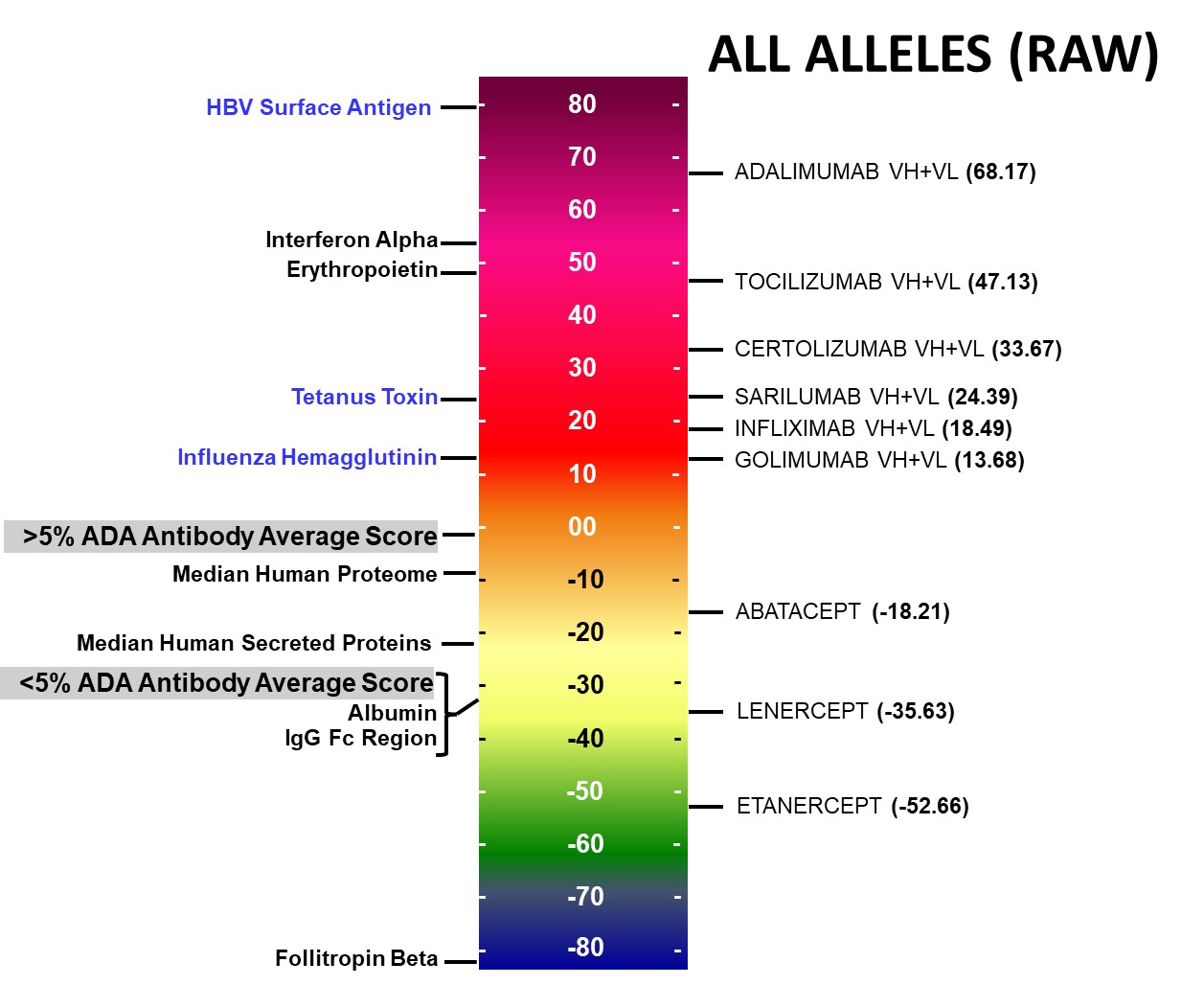


The EpiMatrix Protein Immunogenicity Risk Potential Score represents the aggregate predicted T cell epitope content in each protein, per unit protein length, relative to the expected T cell epitope content in a protein of equivalent length. Proteins with positive scores carry more epitope content than the random expectation, and thereby, increased risk for immunogenic response. Proteins with negative scores carry less epitope content than random expectation, and reduced risk for immunogenic response. These scores are not adjusted for the presence of Tregitopes.
